# Feeding Management in Autistic Children During Early Childhood: A Scoping Review

**DOI:** 10.3390/children12121699

**Published:** 2025-12-16

**Authors:** Noe Jorquera Tobar, Vannia Jara Mella, Daniela Wachholtz Martorell, Samanta Valdés-Thomas, Verónica Vidal Velasco, Evelyn Farías Vargas, Alejandra M. Wiedeman, Marcela Vizcarra Catalán

**Affiliations:** 1 Escuela de Nutrición y Dietética, Facultad de Farmacia, Universidad de Valparaíso, Valparaíso 2340000, Chile; noe.jorquerat@gmail.com (N.J.T.); vannia.jara@uv.cl (V.J.M.); samanta.thomas@uv.cl (S.V.-T.); evelyn.farias@uv.cl (E.F.V.); alejandra.wiedeman@uv.cl (A.M.W.); 2 Escuela de Educación, Facultad de Ciencias Sociales, Universidad de los Andes, Santiago 7620086, Chile; dwachholtz@uandes.cl (D.W.M.); vvidal@uandes.cl (V.V.V.); 3 Research Center for Student Mental Health (ISME), Universidad de los Andes, Santiago 7620086, Chile; 4 Centro de Micro-Bioinnovación, Escuela de Nutrición y Dietética, Universidad de Valparaíso, Valparaíso 2340000, Chile; 5 Centro de Investigación del Comportamiento Alimentario (CEIC), Escuela de Nutrición y Dietética, Facultad de Farmacia, Universidad de Valparaíso, Valparaíso 2340000, Chile

**Keywords:** autism, children, food selectivity, feeding

## Abstract

Autistic children often experience eating difficulties due to sensory processing, food selectivity, and other eating behaviors. As a result, the feeding process can be particularly challenging for caregivers and professionals in healthcare and educational settings. This scoping review describes interventions that address feeding difficulties, focused on improving food acceptance and reducing challenging eating-related behavior in autistic children under 6 years. The review was conducted and reported in accordance with the Preferred Reporting Items for Systematic Reviews and Meta-Analyses (PRISMA-ScR) guidelines. We searched the PubMed, Web of Science (WOS), and PsycINFO databases, as well as manually examined reference lists, to identify relevant articles. Nineteen studies were selected by two independent reviewers for inclusion in the review. Among the selected studies, a variety of effective feeding strategies were categorized into three groups: applied behavior analysis (ABA)-based interventions, a combination of ABA-based strategies with others, and emerging strategies beyond ABA. These interventions have been reported to increase the acceptance of foods and reduce challenging mealtime behaviors of autistic children. Future research should focus on developing comprehensive interventions to improve the quality of life of autistic children, their families, and their communities.

## 1. Introduction

Autism is a neurodevelopmental condition associated with intense or focused interests, distinctive patterns of behavior, communication challenges, and differences in socialization [1]. Globally, autism, found in a proportion of 1 in 100 children, is characterized by differences in sensory processing (such as hearing, smelling, tasting, touching, and seeing), which may result in hyper- or hypo-active behaviors [2]. In autistic children, the brain may process and organize sensory information in either exacerbated or diminished ways, which influences how they respond to their environment [3].

Eating challenges are common in autistic children and are often linked to sensory selectivity and low acceptance of unfamiliar or new foods [4]. In a study of 1443 autistic children, 70.4% had atypical eating behaviors, compared to only 4.8% of neurotypical children [5]. These eating characteristics are explained by 80% of autistic children having difficulties in sensory processing and/or modulation issues that materialize through food selectivity [6]. Such difficulties manifest as food selectivity related to tactile (e.g., refused certain foods because of the temperature [7]), olfactory (e.g., taking vigorous sniffs of unpleasant odors [8]), auditory (e.g., aversion to the sound of chewing), and gustatory (e.g., discomfort with fibrous or soft textures) sensitivities [9]. In general, food selectivity can manifest as food refusal, a limited repertoire of accepted foods, frequent consumption of a single food, excessive intake of certain foods, and selective intake of specific foods [10,11].

Factors related to food selectivity often have a nutritional impact due to inadequate diet quality, raising concerns about children’s health [9]. The prevalence of malnutrition is higher in autistic children compared to the general population [12,13]. In addition, when a diet with poor nutritional value is perpetuated, it can exacerbate sensory sensitivities and further interfere with social interactions and daily routines [14]. There is a relationship between food selectivity and sensory-based difficulties (e.g., taste or smell sensitivities) in autistic children, reflected in their avoidance of fruits and vegetables and in consuming fewer dairy products than children without the diagnosis [15]. These sensory differences are associated with a greater likelihood of persistent patterns of food selectivity that may not change over short periods in autistic children aged 3 to 11 years [16].

These eating difficulties also pose challenges for families and providers. Around 38% of parents report being unable to provide a healthy, nutritious diet for their children, often adapting to their children’s preferred foods to avoid conflicts [17,18]. Therefore, caregivers need tailored guidance from educators and healthcare providers [19]. On the other hand, educators and healthcare providers themselves face barriers in situations related to feeding autistic children. Educators report feeling unprepared due to a lack of time, resources, and institutional support [20], which limits their ability to respond effectively to children’s developmental needs [21]. Similarly, 90% of healthcare providers believe that they require training to offer appropriate care for autistic children [22]. Therefore, feeding management in autistic children presents challenges for families and communities.

Feeding management involves approaches to improving eating and reducing challenging mealtime behaviors in children. Behavioral interventions include applied behavior analysis (ABA), an approach that modifies behavior through diverse techniques and environmental changes [23,24,25]. Sensory interventions involve sensory integration to address food selectivity, considered a manifestation of an altered sensory response [13]. Speech language pathology interventions focus on improving swallowing and communication associated with feeding [26]. Parent training aims at developing parents’/caregivers’ skills and strategies to support feeding at home and modifying behaviors during family mealtime routines [27].

Despite the availability of these approaches, the evidence of their effectiveness, especially in autistic children under 6 years, has not been systematically synthesized. Therefore, this scoping review aims to describe, and systematize feeding management strategies to address eating difficulties in autistic children under 6 years and to inform currently used, evidence-based feeding interventions. A scoping review is suitable for exploring the emerging body of research on feeding management in autistic children during early childhood (e.g., heterogeneity of study designs, characteristics of the feeding intervention) and outcome variables related to food acceptance across diverse samples in the world. Thus, this type of review is useful in identifying gaps in the literature and its implications and suggesting future directions in research to benefit autistic children’s welfare, their families, and their communities.

## 2. Materials and Methods

The guidelines from the Preferred Reporting Items for Systematic Reviews and Meta-Analyses (PRISMA) extension for scoping reviews [28] were used for article verification and selection. The study selection framework used for this review was that of Arksey and O’Malley (2005) [29].

This review uses identity-first (autistic person) language, following current guidelines on autism terminology preferred by communities of autistic people [30] and academic scholars [31].

### 2.1. Identifying Relevant Studies

The systematic literature review was conducted in the following databases: Web of Science (WOS), PubMed, and PsycINFO. The search strategy included the following base terms in PubMed: (“autism” OR “Asperger”) AND (“feeding behavior” OR “intervention” OR “treatment” OR “management” OR “study”) AND (“food selectivity”) AND (“child”). Additionally, the search terms “autism”, “selectivity”, and “behavior” were manually reviewed in the reference lists of the selected articles. The systematic literature review was conducted between 8 and 22 April 2024 and was updated in February 2025.

### 2.2. Study Selection

The inclusion criteria were articles written in English or Spanish; articles published in the last 10 years; application of one or more feeding management strategies to address eating problems in autistic children; children under 6 years; and the dependent variables should include challenging situations during mealtimes and food acceptance (incorporating or trying new foods). Challenging situations during mealtimes are defined in this review as situations that occur with autistic children requiring specific and focused attention from an adult in relation to feeding due to their higher frequency, duration, or intensity compared to other children [19]. The specific definitions considered in the selection of studies are presented in Supplementary Materials S1. Original studies including animal models, drug interventions, conference abstracts, children diagnosed with eating disorders, and children who are tube-fed were excluded.

**Figure 1:**
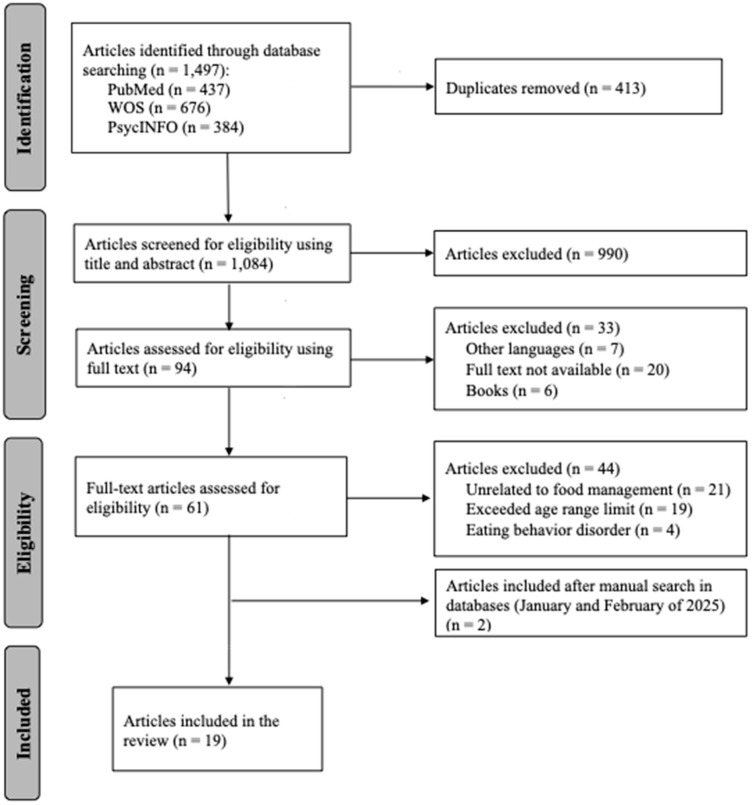
PRISMA flow diagram for scoping review to identify feeding management for autistic children under six years.

The initial search yielded 1497 references that were uploaded to the Mendeley^®^ platform (version 2.112.0), and 413 duplicate articles were removed. One researcher uploaded the file containing non-duplicate articles to the Rayyan^®^ platform (accessed on 1 May 2024) [32], and two independent researchers reviewed the articles based on the inclusion and exclusion criteria. The manual search for reference lists identified 2 additional articles that met the inclusion criteria, as illustrated in Figure 1. For studies reported as a single-case design, the selection was evaluated based on the guidelines of Tate et al., 2016 [33].

## 3. Results

The feeding management strategies were grouped into three categories according to their frequency of use in the studies: (i) only on applied behavior analysis strategies (ABA); (ii) the combination of ABA and other strategies; and (iii) emerging strategies. The synthesis of the studies is presented in Table 1, Table 2 and Table 3.

### 3.1. Studies and Participants’ Characteristics

A total of 19 studies met the inclusion criteria [34,35,36,37,38,39,40,43,44,45,46,47,48,50,51]. The studies were conducted in high-income developed countries such as the USA (n = 12) [35,36,37,40,43,45,46,47,48,50,51], Australia (n = 2) [44,49], and Japan (n = 2) [53]. The study designs were single-case designs (n = 4) [34,36,37,48], randomized clinical trials (n = 3) [35,40,44], and experimental (n = 3) [39,43,47]. The interventions occurred in a therapy room (n = 7) [34,39,47,49,50,51,54], the participant’s home (n = 6) [36,37,38,48,49,53,54], and a hospital or clinic (e.g., feeding disorder clinic) (n = 4) [43,44,46,52]. The sample size ranged from 1 to 68 participants.

Most studies involved male children (n = 18) [34,35,36,38,39,40,43,44,45,46,47,48,49,50,51,52,53,54]. The age range for the children was 2 to 6 years, and for the parents, 20 to 49 years. Four studies reported ethnicity and socioeconomic status [38,46,49,51]. The studies involved European descent (n = 2), African descent (n = 1), South Asian (n = 1), and mixed categories including European and Latin/Hispanic descent (n = 2) and European and Asian descent (n = 1) [38,46,49]. Two studies reported on the socioeconomic status of the families, describing immigrant status without health insurance (n = 1) [49], high socioeconomic status (n = 1) [49], and parental bachelor’s degree (n = 1) [51].

### 3.2. Feeding Managements

The most common feeding management strategies used among the selected studies were prompting [38,44,45,47,49,51], non-removal of the spoon [35,43,47,49], modeling [48,51], lag [36,37], and side deposit (SD) [47,49].

The feeding managements were grouped into three categories according to their frequency of use in the studies: (i) only on applied behavior analysis strategies (ABA); (ii) the combination of ABA and other strategies, and (iii) emerging strategies. Data were summarized and are presented in Table 1, Table 2 and Table 3.

### 3.3. Feeding Management Based on ABA Strategies

The seven studies using ABA strategies (Table 1) [34,35,36,37,38,39,40] included gradual exposure, differential reinforcement of incompatible behavior (DRI), multiple stimulus without replacement (MSWOR), lag intervals, high-probability instructional sequence (HPS), gradual exposure, least-to-most (LTM), response blocking (RB), modified sequential oral sensory approach (M-SOS), non-removal of the spoon (NRS), and continuous interaction.

### 3.4. Feeding Management Based on the Combination of ABA and Other Strategies

Eight studies included feeding management based on a combination of ABA-derived strategies to approach child eating difficulties (Table 2) [43,44,45,46,47,48,49,50,51]. The feeding managements consisted of ABA-derived strategies to approach food acceptance or challenging situations during mealtimes. Three interventions [45,46,51] were aimed at parents and included the use of video modeling or training to implement the feeding strategies with their children [45,51]. Also, one study involved a play-based component aimed at the child [44].

The sensory components were not explicit in the majority of interventions. Some examples included pairing less preferred foods with preferred ones, considering textures that facilitate swallowing [43], and simultaneous presentation, giving food on one side of the mouth due to sensory difficulties, called side deposit (SP) [47,49].

### 3.5. Feeding Management Based on Emerging Strategies

The three studies that involved emerging strategies for feeding management were parent-led debate, sensory strategies, and symbolic play (Table 3) [52,53,54]. The parent-led strategies consisted of a conversation and debate with parents, aimed at identifying factors contributing to nutritional imbalance and considering food selectivity and self-efficacy [52]. The sensory strategies consisted of sensory exploration of foods using symbolic play, in which the child pretends to be a chef enhancing their sensory profile, while decreasing their food selectivity [53,54]. Further details for each strategy regarding feeding management are available in Supplementary Table S1 (ABA-based strategies) and Table S2 (other strategies).

### 3.6. Dependent Variables Employed in the Studies

Several dependent variables describe changes in difficulties or challenging behaviors during mealtimes. Due to the heterogeneous definitions of food acceptance in this review, we have synthesized the terminology and its operationalization in Table 4.

Challenging situations were described as disruptive behavior [45] and emotional responses [54], negative vocalizations [49], packing [38,43,47], inappropriate mealtime behavior [35,40,43,47,49,50,51], disruptive mealtime behavior [37], challenging mealtime behavior [34,46], and difficult mealtime behavior [44].

### 3.7. Frequency and Duration of the Interventions

The frequency of feeding management was used in one study [44]. The authors explained the adherence to sessions as a frequency recommendation for each child, showing an operant condition arm between 7 and 10 sessions for 30–60 min, while for the systematic desensitization arm, the recommendation ranged between 7 and 10 sessions for 30–60 min.

In terms of the dosage of the interventions, the duration of the treatments ranged from one week [47] to three years [53]. Eight studies only mentioned treatment sessions [35,36,37,38,40,46,47,51], one study mentioned treatment sessions and follow-up [43], four studies indicated only intervention time [39,49,53,54], five studies mentioned intervention time and sessions [34,44,48,50,52], and one study did not specify sessions or intervention time [45].

### 3.8. Effectiveness of Feeding Management in Autistic Children and Their Families

Four single-case design (SCD) studies showed changes by using DRI and MSWOR interventions, in which up to 50 foods were included in the diet, accompanied by a reduction in challenging mealtime behaviors [34,36,37,48].

One study reported that one child showed decreased challenging behavior after using NRS, re-distribution, and chaser [43]. The implementation of video modeling aimed at children regarding strategies to eat showed an increase in food acceptance and the incorporation of new textures after teaching the behavior of food acceptance [48]. Another study used video modeling to train mothers on food acceptance by incorporating new foods through a structured meal procedure and reported that two out of three children achieved chewing and bite [50]. To better understand how to apply the procedure, two out of the three mothers needed face-to-face instruction and feedback from the researchers.

The strategy of conversation and debate with parents about the different methods of addressing food selectivity did not find significant changes in the number of foods accepted [52]. In relation to studies with larger samples, Marshall et al. 2015 [44] observed increased diet quality and fewer challenging situations at mealtime in both autistic and neurotypical children after OC plus a prompt-and-reward style or SysD plus play-based intervention.

## 4. Discussion

This review aimed to identify feeding management strategies to address eating-related difficulties and challenging situations in autistic children under 6 years. The most frequent interventions were based on ABA or in combination with other strategies. In most studies, ABA and emerging strategies were found to be effective in increasing food acceptance and addressing challenging situations during mealtimes. Another review also reported a high frequency of ABA strategy use (79%) in a wider age range (6 months–18 years) [59].

Although most studies reported the effectiveness of ABA and other feeding management strategies in this review, these findings should be cautiously analyzed for use during early childhood. ABA interventions have generated controversial opinions due to their undesirable effects on children [23,60,61]. For instance, escape extinction and non-reinforced stimuli have been associated with tantrums, aggressions, noncompliance, or self-harming [62] and could cause trauma [63]. Moreover, it is unclear whether ABA interventions are more effective than other approaches [64].

On the other hand, ABA interventions may be positive for autistic children who have already established eating patterns and exhibit food selectivity. Simultaneous presentation, systematic fading, and high-probability sequence strategies have been related to less challenging situations during mealtimes and increased food acceptance in autistic children [38,47], while respecting their welfare and rights.

Most studies of this review have successfully used feeding management involving the presence of and/or guidance for parents. Modeled videos and family involvement are particularly useful for autistic children with food selectivity [38,51]. Including family-based components can be considered part of more comprehensive interventions in early childhood, aiming for long-term effects [64], as well as for improving malnutrition in autistic children [11].

Most of the studies were conducted in experimental conditions, and few studies were conducted in educational settings or at home, which makes it necessary to assess the effectiveness of feeding management strategies in natural environments. Also, interdisciplinary professional teams could be considered to frame feeding within a multidimensional systemic approach [65].

Although this review describes a diverse array of feeding management strategies to improve food acceptance and other challenging situations during mealtimes in early childhood, it has some limitations. Due to the varied terminology regarding food acceptance, challenging situations during mealtimes, and food management, it is likely that, despite our efforts to include all available evidence, we inadvertently excluded important empirical studies. Also, we excluded investigations in the presence of eating disorders (e.g., avoidant restrictive food intake disorder, ARFID) or medical conditions, such as gastrointestinal disorders (e.g., gastrostomy). Thus, our results may not be representative of the full breadth of feeding management strategies implemented in a heterogeneous population of autistic children in their first years of life. Therefore, a review of studies that involve feeding management for autistic children with eating disorders and/or other concomitant health conditions would be necessary. Those children with one or more health conditions may need treatment in specialized centers due to the complexity of feeding them. For instance, gastrointestinal issues (e.g., food intolerances/allergies, diarrhea/constipation) require treatment beyond food selectivity in autistic children [66]. Also, these health conditions could affect the child’s ability to eat and, in turn, the progression of the intervention.

While other reviews have been focused on the nutritional intake in autistic children with food selectivity [49,67], our review highlights how to approach feeding children in the key period of early childhood and the gaps related to it. Also, we highlight the need for multicomponent interventions that account for the context and real-life needs of autistic children. From a global perspective, more research is necessary in different regions of the world, such as Asia, Africa, South America, Oceania, and Europe, and where culture and context differ from those in developed countries, where most studies have been conducted.

According to our review, there is a need for studies that use standardized outcome measures of food acceptance and that characterize sociodemographic factors such as cultural background, socioeconomic status, and race/ethnicity. In terms of study designs, longitudinal studies with larger samples are required to assess the dosage and the long-term evolution of interventions to generalize findings. Another review also discussed the need to address these gaps [24], as well as studies involving multicomponent interventions including families, educators, and the child.

## 5. Conclusions

Among a range of feeding management strategies reported, ABA strategies, whether alone or in combination with other approaches, are the most commonly implemented to address food acceptance and mealtime behaviors. However, multicomponent interventions that support children and caregivers in home and school environments are promising in a framework of children’s rights, development, and welfare. Further research requires study designs that clarify the dosage of the intervention to increase long-term food acceptance and challenging situations during mealtimes, as well as research conducted in diverse samples beyond high-income countries that could generalize findings. Collaborative involvement of families, healthcare providers, and educators is essential to support optimal nutrition and to help autistic children learn to eat at a comfortable pace according to their needs.

## Figures and Tables

**Table 1:** Synthesis of selected studies involving feeding management regarding ABA-derived strategies.

Author, Year	Country	Sample Size, Sex, and Sociodemographics	Setting	Study Design	Feeding Management	Number of Sessions	Dependent Variable (Food Acceptance, Challenging Situations ^1^)	Results
Tanner & Andreone, 2015 [34]	Canada	One boy of 3 years	Therapy room at autism center and ABA therapy room	Single-case design	A 12-step gradual exposure of target food, DRI, and MSWOR (array of four to six pictures, which represented preferred items and activities)	100 sessions in 9 months	i. Independent oral consumption of target foods during treatment measured by bites ii. Independent oral consumption of target foods outside of treatment iii. Challenging mealtime behavior	Food consumption increased, challenging mealtime behavior decreased, and the child remained at the table during meals in settings and with people outside the treatment room and at home.
Peterson et al., 2016 [35]	USA	Six boys between 4 and 6 years	Rooms with adjacent one-way observation panels	Randomized clinical study	It was divided into three groups in duos, applying ABA and the other M-SOS. They were complemented by NRS with continuous interaction	Between 12 and 19 sessions	i. Food acceptance ii. Inappropriate mealtime behavior iii. Mouth clean	In M-SOS, food acceptance increased after switching to ABA. Adding continuous interaction to EE reduced inappropriate mealtime behaviors.
Silbaugh & Falcomata, 2016 [36]	USA	One boy of 4 years	Spare bedroom of a house	Single-case design	Lag 0 (A, every independent bite consumed resulted in removal of the plate and access to highly preferred toys). Lag 1 (B, access to highly preferred toys was provided on variant consumption)	30 sessions 2 to 3 times a week	i. Variability of food consumption ^2^ ii. Mealtime behavior	Food consumption was achieved independently and there was no challenging behavior at mealtimes in both treatments.
Silbaugh et al., 2017 [37]	USA	One girl of 3 years	Home	Single-case design	Lag 1 (lag 1/toys, lag 1 /toys/LTM/RB more EE and lag 1/RB) and lag 2 (lag 2/RB)	35 sessions	i. Independent variant consumption ii. Variability ^2^ iii. Disruptive mealtime behavior	Consumption of food remained high and disruptive behavior decreased in sessions where toys were not available and in lag 2/RB.
Silbaugh & Swinnea, 2018 [38]	USA	One girl of 5 years (Hispanic and Caucasian) and two boys (both Caucasian)	Kitchen at home and spare classroom at school	Case report	HPS with EBPs (levels 1 to 7: contact compliance with prompt model and levels 8 and 9: bite, chewing, and swallowing compliance)	4 to 39 sessions	i. Mealtime behavior (Compliance with HP mealtime demands) ii. Food acceptance (Compliance with LP mealtime demands) iii. Packing ^3^	Both children increased their food intake and decreased inappropriate eating behavior and disruptive behavior. One of them increased the consumption of more textured foods.
Kim et al., 2018 [39]	South Korea	27 kids (24 boys and 3 girls between 2 and 5 years) and their parents	Treatment room	Experimental design	Program of gradual exposure to vegetable consumption. The exposure group comprised 13 and the control group 12 individuals	6 months	i. Vegetable consumption ii. Sensory stimulation	There were significant differences between the exposure and control groups in vegetable consumption but not in touch or taste.
Peterson et al., 2019 [40]	USA	Six boys between 3 and 6 years	Observation room	Randomized clinical study	Three groups were divided into pairs, with one group receiving ABA and the other a wait-list control group	160 sessions	i. Independent acceptance ii. Mouth clean iii. Inappropriate mealtime behavior	Independent acceptance of target, novel, and non-preferred foods increased for the ABA and wait-list control groups. Inappropriate mealtime behavior varied for both groups.

^1^ Challenging situations: In this review, it refers to situations that occur with autistic children requiring specific and focused attention from an adult in relation to feeding due to their higher frequency, duration, or intensity compared to other children [19]. We considered challenging situations described in the literature, such as disruptive behaviors, dysregulation episodes, challenging mealtime behavior, packing, or inappropriate mealtime behavior. ^2^ Variability of foods: diversity and breadth of foods that a person consumes [41]. ^3^ Packing: defined as holding or keeping solids or liquids without swallowing them [42]. Non-Removal of the Spoon (NRS), Multiple Stimulus Without Replacement (MSWOR), Differential Reinforcement of Incompatible Behavior (DRI), Modified Sequential Oral Sensory (M-SOS), Escape Extinction (EE), Least-to-Most (LTM), Response Blocking (RB), Applied Behavior Analysis (ABA), High-Probability Instructional Sequence (HPS), Evidence-based Practices (EBPs).

**Table 2:** Synthesis of selected studies involving feeding management regarding ABA-derived strategies in combination with other strategies.

Author, Year	Country	Sample Size, Sex, and Sociodemographics	Setting	Study Design	Feeding Management	Number of Sessions	Dependent Variable (Food Acceptance, Challenging Situations ^1^)	Results
Levin et al., 2014 [43]	USA	Two boys and one girl, both 4 years	Feeding disorder clinic at a major medical center	Experimental design	The modification of consistencies with re-distribution, swallow facilitation, NRS, and a chaser in mealtimes	60 to 90 sessions. Six-month follow-up	i. Bite acceptance ii. Mouth clean iii. Packing ^2^ iv. Inappropriate mealtime behavior	One increased food consumption, decreased food packaging and inappropriate mealtime behavior ^3^.
Marshall et al., 2015 [44]	Australia	68 (50 boys and 18 girls between 2 and 6 years)	Tertiary pediatric hospital	Randomized clinical trial	Individuals were assigned into the AS or NMC group, in which the intervention could be OC plus a prompt-and-reward style or SysD plus a play-based intervention	10 sessions in 10 weeks. Intensive 10 sessions in 1 week. 3-month follow-up	i. Dietary variety ii. Difficult mealtime behaviors	There was a greater increase in fruit and vegetable consumption and a reduction in difficult mealtime behaviors in the OC group than in the SysD group. Results in the AS group improved in diet quality, and the NMC group improved in diet variety.
Barnhill et al., 2016 [45]	USA	One girl of 2 years	Small office with one door but no windows	Case report	Parent training, shaping, DRA, prompting, and EE	Not specified	i. Food acceptance ii. Bite acceptance iii. Bite consumption iv. Mealtime behavior v. Disruptive behavior	Acceptance and food consumption increased rapidly. There were generalized skills given to the parents, grandmother, and other caregivers.
Muldoon & Cosbey, 2018 [46]	USA	Three boys between 3 and 5 years (one Indian, one, black and one white Hispanic) and four (three mothers and one father) parents = seven	Small hospital outpatient clinic in a rehabilitation hospital	Pilot study	Easing Anxiety Together with Understanding and Perseverance (EAT-UP)	36 sessions	i. Challenging mealtime behaviors ii. Food acceptance (less preferred food)	Increased acceptance and variety of foods, decrease in challenging behavior and amount of problem behavior with caregivers at mealtimes.
Whipple et al., 2020 [47]	USA	One boy of 4 years	Treatment room	Experimental design	Systematic fading of simultaneous presentation. If still did not accept the bite, the therapist implemented DRA, also FP + SD and NRS	47 sessions are shown in graphs, but not specified in the written text	i. Expels ii. Inappropriate mealtime behavior iii. Packing ^2^ iv. Meal duration	Inappropriate behavior decreased with simultaneous presentation of two chocolate chips. Packing ^2^ remained at 0% with 1.5 chips, meal duration and inappropriate behavior remained low.
Hillman, 2019 [48]	USA	Two boys and one girl (3–4 years)	Home	Single-case design	Video modeling or video modeling plus reinforcement condition all performed by the principal investigator	32 sessions in 5 months	i. Food acceptance ii. Food consumption	All three children incorporated new foods with the use of video modeling, and one of them required additional reinforcement.
Taylor, 2020 [49]	Australia	Two boys of 0-5 years (One Asian Caucasian and one Caucasian)	Dining room and therapy room	Clinical case	Differential attention and contingent access, NRS, FP, and SD	3 weeks	i. Food acceptance ii. Mouth clean iii. Inappropriate mealtime behavior iv. Negative vocalizations	Both children increased their food intake and decreased inappropriate feeding behavior and negative vocalizations. One learned to feed himself and eliminated dependence on formula and baby bottles.
Gover et al., 2023 [50]	USA	Three kids (Two boys and one girl between 4 and 6 years) and three mothers = six	Small treatment room	Design of non-concurrent multiple baseline	Video modeling prompting to train parents on structured meal procedures. The child was presented with a board that visually displays the different response topographies (e.g., touch, smell, taste), giving a corresponding behavior for the application of shaping, such as contingent reinforcement and differential reinforcement	Between 8 and 33 sessions from 3 weeks to 4 months	i. Bite/chewing acceptance ii. Inappropriate mealtime behavior	All three children increased their food consumption and without challenging behavior with family members.
Clark et al., 2020 [51]	USA	Three boys between 3 and 6 years and three mothers between 33 and 38 years	Treatment room at a university	Non-concurrent multiple-baseline design	Video modeling prompting to train parents on structured meal procedures	18 sessions	i. Bite acceptance ii. Parent performance	Two children increased bite acceptance and one child did not exhibit an increase. Two of the three mothers needed feedback and prompting in vivo.

^1^ Challenging situations: These refer to situations that occur with autistic children requiring specific and focused attention from an adult in relation to feeding due to their higher frequency, duration, or intensity compared to other children [19]. We considered challenging situations described in the literature, such as disruptive behaviors, dysregulation episodes, challenging mealtime behaviors, packing, or inappropriate mealtime behaviors. ^2^ Packing: defined as holding or keeping solids or liquids without swallowing them [42]. ^3^ One of the two children was dependent on a gastrostomy; therefore, we excluded the results of this child from the analysis. Non-Removal of the Spoon (NRS), Autism Spectrum (AS), No Medical Conditions (NMC), Operant Conditioning (OC), Systematic Desensitization (SysD), Differential Reinforcement of Alternative Behavior (DRA), Escape Extinction (EE), Finger Prompt (FP), Side Deposit (SD).

**Table 3:** Synthesis of selected studies involving feeding management regarding emerging strategies.

Author, Year	Country	Sample Size, Sex, and Sociodemographics	Setting	Study Design	Feeding Management	Number of Sessions	Dependent Variable (Food Acceptance, Challenging Situations ^1^)	Results
Miyajima et al., 2017 [52]	Japan	23 parents between 20 and 49 years and 23 kids (18 boys and 5 girls) = 46	Developmental support centers in Japan	Self-controlled trial	Parent-led debate to identify underlying factors of nutritional imbalance and approaches to dietary selectivity and self-efficacy	Two sessions and two debates for 2 months	i. Degree of self-efficacy of the parents ii. Incorporation and acceptance of new foods	There was no significant change in the number of foods accepted. Self-efficacy and knowledge of approaches improved. Parents in small groups were able to familiarize themselves with food preferences and what was best for their family dynamic.
Yamane et al., 2019 [53]	Japan	30 boys and 10 girls between 3 and 6 years	Home	Retrospective analysis	Administration of a diet based on the developmental assessment with the characteristics of each food for each group: one crunchy, two visual and three habitual	1 to 3 years	i. Food incorporation ii. Sensory profile	Group 1 began to eat vegetables, fish, meat, and tofu. Some children in group 1 and 2 began to prefer foods of particular shapes. No significant differences were observed in sensory assessment among the groups.
Oliveira & Souza, 2022 [54]	Brazil	One boy of 5 years	Specific room for sensory integration and home	Experience report	Symbolic play, giving new meaning to the everyday food scenario, through characters and real and non-real foods at Sensory Integration Therapy	1 year	i. Food acceptance ii. Sensory profile iii. Behavioral and emotional responses	The therapist encouraged the preparation of a menu with a list of ingredients and the final product, increasing their food consumption and participation at mealtimes.

^1^ Challenging situations: These refer to situations that occur with autistic children requiring specific and focused attention from an adult in relation to feeding due to their higher frequency, duration, or intensity compared to other children [19]. We considered challenging situations described in the literature, such as disruptive behaviors, dysregulation episodes, challenging mealtime behaviors, packing, or inappropriate mealtime behaviors.

**Table 4:** Definitions and terms used to describe food acceptance in the selected studies.

Terminology	Authors	Operationalization of Food Acceptance
Food acceptance	Peterson et al., 2016 [35]	When the child used the utensil or their fingers to put the entire bite of food in their mouth within 8 s of presentation, not including placement of the bite in the mouth during re-presentation.
	Silbaugh & Swinnea, 2018 [38]	In the context of the mealtime demands of the intervention, it was defined depending on the child’s needs based on accepting, chewing and swallowing the bite of non-preferred foods, checked by a clean mouth (5 s or 30 s)
	Levin et al., 2014 [43]	The entire bite entered the child’s mouth within 5 s of Presentation.
	Hillman, 2019 [48]	When the child opens the mouth and places a bite of food into the mouth using hands or eating with utensils within 10 s after the presentation of the food and verbal prompt.
	Taylor, 2020 [49]	When the child’s entire bolus, except for an amount smaller than a pea, passed the plane of the lips into the mouth for the first time at any time during each bite presentation.
	Muldoon & Cosbey, 2018 [46]	Food Frequency Questionnaire (adapted from Harvard T.H. Chan School of Public Health, 2012 [55]) and 24 h food recall (adapted from Lukens, 2008 [56]).
	Miyajima et al., 2017 [52]	The changes in the eating patterns of the autistic children: (i) the number of foods that the child chose to eat (47 items) and (ii) the parents’ subjective view of the degree of dietary imbalance (VAS; 0–100 points [57]).
	Oliveira & Souza, 2022 [54]	Not mentioned.
Independent acceptance	Peterson et al., 2019 [40]	If the child picked up the spoon, fork, or bite of food and deposited the entire bite, except for food the size of a grain of rice or smaller, into his or her mouth within 8 s of presentation.
Independent consumption of food/independent oral consumption of a target food	Tanner & Andreone, 2015 [34]	If the child eats an entire spoonful or spoonful-sized piece of a previously refused food, without the use of any physical prompting.
Independent variant consumption	Silbaugh et al., 2017 [37]	Actual or attempted independent consumption of a food that differed from the immediately preceding food consumed within the session.
Bite/chewing acceptance	Gover et al., 2023 [50]	If the child engagement with the food was scored, the corresponding consequences were delivered, and that trial was complete.
Bite acceptance	Barnhill et al., 2016 [45]	Opening the mouth 1.3 cm or wider within 5 s of the bite presentation and allowing food placement in the mouth and placing food in the mouth within 5 s of bite presentation.
	Levin et al., 2014 [43]	The entire bite entered the child’s mouth within 5 s of presentation.
	Clark et al., 2020 [51]	As the child actively lifting the feeding spoon themselves and depositing the entire bolus past the plane of the lips, and refusal, which we defined as the child not depositing the bolus past the plane of the lips.
Expels	Whipple et al., 2020 [47]	A previously accepted piece of food larger than the size of a pea was present outside of the mouth.
Bite consumption	Barnhill et al., 2016 [45]	Swallows bite, and the mouth is clean of food.
Mouth clean	Peterson et al., 2016 [35]	When there was no food larger than a grain of rice in the child’s mouth 30 s after the entire bite entered the mouth, excluding the absence of food in the mouth as a result of expulsion (i.e., spitting out the bite).
	Peterson et al., 2019 [40]	
	Taylor, 2020 [49]	Product measure of swallowing; no food larger than the size of a pea in mouth at a 30 s “show me” check unless absence of food was due to expulsion.
	Levin et al., 2014 [43]	The absence of food was not due to expulsion, the feeder provided praise, and the observer scored the mouth clean. The definition for one child accounts for the small size of the presented bite.
Vegetable consumption	Kim et al., 2018 [39]	Clearing the mouth within the first 30 s after acceptance. Touch was defined as at least a single tactile point of contact with the vegetable pieces.
Food consumption	Hillman, 2019 [48]	
Food consumption with different sensory profile	Kim et al., 2018 [39]	When the child touches at least a single tactile point of contact with the vegetable pieces and taste was defined as putting the pieces in the mouth without swallowing.
	Yamane et al., 2019 [53]	JSI-R (Japanese Sensory Inventory Revised) performed by parents for sensory assessment [58].
	Oliveira & Souza, 2022 [54]	Analysis of the items that directly influenced the diet, namely the visual, vestibular, proprioceptive, tactile, oral (olfactory and gustatory) system, and the sensory sensitivity and oral sensory sensitivity quadrants.
Variability of food consumption	Silbaugh & Falcomata, 2016 [36]	It was based on visual analysis of the variability data. Within the session, the variety of food consumed was measured concurrently.
Dietary variety	Marshall et al., 2015 [44]	Number of foods consumed and accepted based on the management applied: (i) OC arm included offering three foods per session. (ii) SysD arm included offering 10 foods per session, offering a range of textures during each session.

## Data Availability

The original contributions presented in this study are included in the article/Supplementary Material. Further inquiries can be directed to the corresponding author.

## Supplementary Materials

The following supporting information can be downloaded at https://www.mdpi.com/article/10.3390/children12121699/s1, Supplementary Material S1: Definitions for the selection of studies; Table S1: Definitions of food strategies based on the ABA method; Table S2: Definition of feeding management within other strategies applied in the results [68,69,70,71,72,73,74,75,76,77,78,79,80,81,82,83,84,85,86,87,88,89,90,91,92,93,94,95,96,97,98].
