# Supplementary material for: Feeding Management in Autistic Children During Early Childhood: A Scoping Review
Source: Children (Basel). 2025 Dec 16;12(12):1699. doi: 10.3390/children12121699 (PMC12732215; doi:10.3390/children12121699)
Supplement: Supplementary file 1 [file children-12-01699-s001.zip › children-3992473-supplementary.pdf]

SUPPLEMENTARY MATERIAL S1

The following definitions were considered in the selection of studies:

- Feeding management: Defined as strategies aimed at improving feeding problems or challenging situations related to feeding. These strategies may be individual, family-based, community-oriented, and environment-related, among others.

- Challenging situation: Refers to situations occurring with autistic children that require specific and focused attention from an adult in relation to feeding due to higher frequency, duration, or intensity compared to other children (Ministerio de Educación de Chile, 2023). We considered the following episodes:

    - Mealtime behaviors: Refers to behaviors exhibited during meals that may include attitudes toward food (e.g., food refusal), the amount of food consumed, table behavior (e.g., spitting out food), or any behavior that influences the interaction with food or the people the child is eating (Alonzo-Castillo et al., 2023).

    - Disruptive behavior: Described as severe ways of normative misbehavior during mealtimes, including tantrums, aggressions or noncompliance, or self-injurious/self-harming behaviors (Wakschlag et al., 2009).

- Food acceptance/Food acceptability: Refers to how a child accepts food, for example, when a child interacts with the food offered during an intervention by biting, tasting, or swallowing it. This definition includes when the child introduces and accepts

new foods or improves food variability or diet variety, meaning that the child introduces foods that are not part of their diet (Dovey et al., 2009). This is assessed based on different sensory factors, including flavor, texture, smell, appearance, and previous experiences with food (Pliner & Stallberg-White, 2000).

Supplementary Table S1. Definitions of food strategies based on ABA method

**Escape Extinction (EE)**	It is a procedure that is implemented when a child's feeding problem is presumed to be maintained by negative reinforcement, escape, or avoidance. The demand to eat is not allowed (Bachmeyer, 2009).
**Non-Removal of the Spoon (NRS)**	This is an example of an EE procedure, involving placing the spoon in front of the child's mouth until the child accepts the bite, preventing escape or avoiding the bite (Bachmeyer, 2009).
**Differential reinforcement**	It is a process that focuses on reinforcing desirable behaviors while reducing or extinguishing undesired ones; it can be positive or negative reinforcement (Cooper et al., 2019).
**Differential Reinforcement of Alternative behavior (DRA)**	Involves reinforcing a desirable child´s behavior (e.g., accepting food or swallowing bites of food) while retaining reinforcement for an undesirable behavior through contingent access to preferred foods (e.g., format, and flavor) (Bachmeyer, 2009).
**Differential Reinforcement of Incompatible behavior (DRI)**	Refers to DRA procedures in which the alternative response is selected because it is not possible to simultaneously engage in the alternative response and the undesired behavior. In other words, the alternative response is incompatible with also engaging in the undesired or challenging behavior (Stabel et al., 2013).
**Reinforcement and Reinforcement condition**	A relationship between two events in the environment, one of which is a behavior (response) and the other of which is an event or consequence that follows the response; the behavior increases or maintains its rate because of the consequence (Taylor, 2022).
**Lag**	It refers to the time interval between repeated study sessions or repetitions of the same material, improving long-term memory (Kahana & Howard, 2005).

**Operant Conditioning (OC)**	It is a learning method that uses rewards and punishments to modify behavior. Behavior that is rewarded is more likely to be repeated, while behavior that is punished is less likely to be repeated (Stadoon & Cerutti, 2003).
**Systematic Desensitization (SyD)**	It is a type of behavioral therapy that aims to change the way a person responds to objects, as well as to people or situations that trigger feelings of fear and anxiety (Dubord, 2011).
**Fading and Systematic fading**	It refers to the decrease in the level of assistance needed to complete a task or activity. When a skill is taught, the overall goal is for the student to eventually engage in the skill independently (Cooper et al., 2019).
**High Probability instructional Sequence (HPS)**	It is a sequence of high-implication instructions, issuing several instructions that are likely to be complied with immediately prior to the issuance of an instruction that is unlikely to be complied with (Bullock & Normand, 2006).
**Multiple Stimulus Without Replacement (MSWOR)**	It involves the issuance of several instructions that are likely to be complied with immediately before the issuance of an instruction that the participant is unlikely to comply with. It is praised or giving some other potential reinforcer after each instance of compliance (Daly et al., 2009).
**Response Blocking (RB)**	It is the physical prevention of maladaptive behavior, such as self-injury (e.g., eye gouging), aggression, throwing objects, loud clapping, and mouth use. In general, a clearly visible motor response is required for response blocking to be used (Kearney et al., 2005).
**Prompt**	This occurs when parents or therapists are involved in encouraging the desired response. Depending on the person and the skills being taught, a hierarchy of prompting may be used from most intrusive to least intrusive and teaching in a manner referred to as "error-free" (Godby et al., 1987).

**Finger prompt (FP)**	It involves a professional inserting the forefinger into a child's mouth along the upper gum line (Borrero et al., 2013).
**Least to most (LTM) prompting**	It involves starting with minimal assistance and gradually increasing the level of stimulation according to the individual's needs (Libby et al., 2008).
**Modified Sequential Oral Sensory Approach (M-SOS)**	**The authors modified the term SOS method following the study by Toomey, 2007.** It is a transdisciplinary approach to the assessment and treatment of feeding difficulties. It addresses the physical reasons for the problems and ensures that they are medically addressed. It addresses the child's nutritional deficits and makes treatment recommendations, as well as for the development of sensory, motor, oral-motor, and cognitive skills (Benson et al., 2013).
**Gradual exposure**	It constructs an exposure hierarchy, in which objects, activities, or situations are ranked according to their difficulty. It starts with exposures of mild or moderate difficulty and then progresses to more difficult ones (APA Div. 12 (Society of Clinical Psychology), 2025).
**Continuous interaction**	It involves tracking and recording each occurrence of a behavior during the observation period. This method ensures that no instance of the behavior goes unnoticed, allowing a detailed analysis of its frequency, duration, and intensity (Cooper et al., 2019).
**Contingent access**	It refers to the degree to which knowledge of one event reduces uncertainty about another event by positive reinforcement (Bachmeyer, 2009).
**Contingent reinforcement**	It is a strategy in which reinforcement is applied only after the desired behavior is exhibited (Hulac et al., 2016).
**Shaping**	The contingencies of reward are progressively altered to successively approach a desired behavior. The motivational state influences the child to approach or avoid events that are rewarding or punitive (Sutton & Barto, 2015).

Supplementary Table S2. Definition of feeding management within experimental designs applied in the results.

**Structure meal procedures**	It consists of environmental manipulations that provide structure to the food block, which can result in appropriate eating behaviors. Its main component is the differential reinforcement of alternative behaviors for snack acceptance (Clark, 2019).
**Differential attention**	Positive attention to the child's appropriate behaviors and ignoring inappropriate behaviors (Pemberton et al., 2013).
**Side deposit**	A strategy where food is placed precisely on one side of the child's mouth to facilitate swallowing or chewing (Taylor, 2020).
**Simultaneous presentation**	It is the presentation of a more preferred food with a less preferred food to increase food preferences, in relation to following a less preferred food with a more preferred food as a result of taste-flavor learning (Piazza et al., 2002).
**Chaser**	A method that uses normal eating to help swallow more difficult food textures and consistencies is to follow bites of solids with liquids or to follow a difficult food with a bite of a softer food (Yamane et al., 2020).
**Swallow facilitation**	It involves physical or behavioral interventions designed to facilitate the swallowing process (Yamane et al., 2020).
**Re-distribution**	It involves removing the packaged food from the child's mouth and then effectively repositioning it on the tongue or other optimal location within the mouth to facilitate swallowing (Gulotta et al., 2005).
**Symbolic play**	It is the ability to symbolize, in other words, to create mental situations and combine real facts with imaginative facts. It consists of creating real or imaginary situations, imitating characters that are not present in the game (González-Villavicencio et al., 2022).

**Play-based intervention**	It is designed to enhance social-emotional, physical, language, and cognitive development through guided interactive play. It uses strategies that include modeling, verbal redirection, reinforcement, and indirect instruction to sustain and encourage the child's play activities (Miller, 2017).
**Modeling and Video modeling**	The process in which one or more individuals or other entities serve as examples (models) that a child will emulate. These models are often parents, other adults, or other children, but they may also be symbolic (e.g., a book or television character) (American Psychological Association, 2023).
**Easing Anxiety Together with Understanding and Perseverance (EAT-UP)**	It is a multicomponent intervention program for mealtime that is implemented by parents and designed by a speech language pathologist and an occupational therapist with the goal of helping other professionals and parents improve the acceptance of less preferred foods by their children and reduce disruptive behaviors during meals (Muldoon & Cosbey, 2018).

References

1. Alonzo-Castillo, T., Lugo-Marín, J., Robles, M., Rossich, R., Gallego, L., González, M., Setién-Ramos, I., Martínez-Ramírez, M., Ramos-Quiroga, J. A., & Gisbert-Gustemps, L. (2023). Trastorno del espectro autista: impacto de una estrategia de formación en línea en los conocimientos del personal sanitario de un hospital de tercer nivel. Revista de Neurología, 78(01), 1. https://doi.org/10.33588/rn.7801.2023244

2. American Psychological Association (2023). Modeling. APA Dictionary of Psychology.

3. APA Div. 12 (Society of Clinical Psychology). (2023). What Is Exposure Therapy? Clinical Practice Guideline For The Treatment Of Posttraumatic Stress Disorder (PTSD) In Adults. https://www.apa.org/ptsd-guideline/patients-and-families/exposure-therapy

4. Bachmeyer, M. H. (2009). Treatment of selective and inadequate food intake in children: A review and practical guide. Behavior Analysis In Practice, 2(1), 43-50. https://doi.org/10.1007/bf03391736

5. Daly EJ, Wells NJ, Swanger-Gagné MS, Carr JE, Kunz GM, Taylor AM. Evaluation of the multiple-stimulus without replacement preference assessment method using activities as stimuli. Journal of Applied Behavior Analysis. 2009 Sep;42(3):563–74. http://doi.org/10.1901/jaba.2009.42-563.

6. Benson, J. D., Parke, C. S., Gannon, C., & Muñoz, D. (2013). A retrospective analysis of the sequential oral sensory feeding approach in children with feeding difficulties. Journal Of Occupational Therapy Schools & Early Intervention, 6(4), 289-300. https://doi.org/10.1080/19411243.2013.860758

7. Borrero, C. S. W., Schlereth, G. J., Rubio, E. K., & Taylor, T. (2013). A comparison of two physical guidance procedures in the treatment of pediatric food refusal. Behavioral Interventions, 28(4), 261-280. https://doi.org/10.1002/bin.1373

8. Bullock C, Normand MP. The effects of a high-probability instruction sequence and response-independent reinforcer delivery on child compliance. Journal of Applied Behavior Analysis. 2006 Dec;39(4):495–9. http://doi.org/10.1901/jaba.2006.115-05.

9. Clark, R. (2019). "Training caregivers to implement a structured meal protocol to decrease food selectivity among young children with autism". Theses and Dissertations. 101. https://repository.fit.edu/etd/101

10. Cooper, J. O., Heron, T. E., & Heward, W. L. (2019). Applied Behavior Analysis (3er ed.). NJ: Pearson Education

11. Dovey, T. M., Staples, P. A., Gibson, E. L., & Halford, J. C. (2007). Food neophobia and 'picky/fussy' eating in children: A review. Appetite, 50(2-3), 181-193. https://doi.org/10.1016/j.appet.2007.09.009

12. Dubord G. Part 12. Systematic desensitization. Canadian Family Physician. 2011 Nov;57(11):1299.

13. Godby, S., Gast, D. L., & Wolery, M. (1987). A comparison of time delay and system of least prompts in teaching object identification. Research In Developmental Disabilities, 8(2), 283-305. https://doi.org/10.1016/0891-4222(87)90009-6

14. González-Villavicencio, J. L., Vele-Caymayo, D. M., Tapia-Brito, D. Y., Salgado-Oviedo, P. B. (2022). El juego simbólico como estrategia para el desarrollo psicomotriz de los niños. Polo del Conocimiento, 7(2):1815–25. https://doi.org/10.23857/pc.v7i2.3682

15. Gulotta, C. S., Piazza, C. C., Patel, M. R., & Layer, S. A. (2005). Using food redistribution to reduce packing in children with severe food refusal. Journal Of Applied Behavior Analysis, 38(1), 39-50. https://doi.org/10.1901/jaba.2005.168-03

16. Hulac, D., Benson, N., Nesmith, M. C., & Shervey, S. W. (2016). Using Variable Interval Reinforcement Schedules to Support Students in the Classroom: An Introduction With Illustrative Examples. Journal Of Educational Research And Practice, 6(1). https://doi.org/10.5590/jerap.2016.06.1.06

17. Kahana MJ, Howard MW. Spacing and lag effects in free recall of pure lists. Psychonomic Bulletin & Review. 2005 Feb;12(1):159–64. http://doi.org/10.3758/bf03196362.

18. Kearney C, Linning L, Alvarez K. Response blocking. In: Encyclopedia of Behavior Modification and Cognitive Behavior Therapy. Vol. 3. SAGE Publications, Inc.; 2005. p. 1001-2. https://doi.org/10.4135/9781412950534

19. Libby, M. E., Weiss, J. S., Bancroft, S., & Ahearn, W. H. (2008). A comparison of most-to-least and least-to-most prompting on the acquisition of solitary play skills. Behavior Analysis In Practice, 1(1), 37-43. https://doi.org/10.1007/bf03391719

20. Miller, L. J. (2017). Creating a common terminology for play behavior to increase cross-disciplinary research. Learning & Behavior, 45(4), 330-334. https://doi.org/10.3758/s13420-017-0286-x

21. Ministerio de Educación de Chile (2023). Orientaciones para la atención de niños y niñas con Trastorno del Espectro Autista (TEA). Santiago, Chile: Ministerio de Educación. p. 13. https://parvularia.mineduc.cl/wp-content/uploads/2023/10/Orientaciones-para-TEA-16-oct.pdf

22. Muldoon, D., & Cosbey, J. (2018). A family-centered feeding intervention to promote food acceptance and decrease challenging behaviors in children with ASD: Report of follow-up data on a train-the-trainer model using EAT-UP. American Journal of Speech-Language Pathology, 27(1), 278–287. https://doi.org/10.1044/2017_AJSLP-17-0105

23. Pemberton, J. R., Borrego, J., & Sherman, S. (2012). Differential attention as a mechanism of change in parent–child interaction therapy: Support from time-series analysis. Journal Of Psychopathology And Behavioral Assessment, 35(1), 35-44. https://doi.org/10.1007/s10862-012-9312-7

24. Piazza, C. C., Patel, M. R., Santana, C. M., Goh, H., Delia, M. D., & Lancaster, B. M. (2002). An evaluation of simultaneous and sequential presentation of preferred and nonpreferred food to treat food selectivity. Journal Of Applied Behavior Analy

25. Pliner, P., & Stallberg-White, C. (2000). "Pass the ketchup, please": familiar flavors increase children's willingness to taste novel foods. Appetite, 34(1), 95-103. https://doi.org/10.1006/appe.1999.0290

26. Stabel, A., Kroeger-Geoppinger, K., McCullagh, J., Weiss, D., McCullagh, J., Schneider, N., Newman, D. B., Schultz-Krohn, W., Volkmar, F. R., Glennon, T. J., Austin, S., Winterling, V., Spencer, E., Califano, C., Hansen, R., El-Fishawy, P., Scahill, L. D., Shtayermman, O., Benson, B. A., … Califano, C. (2013). Differential Reinforcement Procedures of Alternative Behavior (DRA/DRAlt) of Incompatible Behavior (DRI). In Encyclopedia of Autism Spectrum Disorders (pp. 954–958). Springer New York. https://doi.org/10.1007/978-1-4419-1698-3_1904

27. Staddon JER, Cerutti DT. Operant conditioning. Annual Review of Psychology. 2003 Jun 10;54(1):115–44. http://doi.org/10.1146/annurev.psych.54.101601.145124

28. Sutton, R., & Barto, A. (2015). Reinforcement Learning: An introduction (Second edition). The MIT Press.

29. Taylor, S. S. (2022). Reinforcement. In Reinforcement. Routledge. https://doi.org/10.4324/9780367198459-REPRW174-1

30. Taylor, T. (2020). Side deposit with regular texture food for clinical cases in-home. Journal of Pediatric Psychology, 45(4), 399–410. https://doi.org/10.1093/jpepsy/jsaa00

31. Toomey, K. (2007, July). An introduction to the SOS approach to feeding. Pediatric Feeding and Dysphagia Newsletter, 8(1), 2–10.

32. Wakschlag, L. S., Tolan, P. H., & Leventhal, B. L. (2009). Research Review: 'Ain't misbehavin': Towards a developmentally-specified nosology for preschool disruptive behavior. Journal Of Child Psychology And Psychiatry, 51(1), 3-22. https://doi.org/10.1111/j.1469-7610.2009.02184.x

33. Yamane, K., Fujii, Y., & Hijikata, N. (2020). Support and development of autistic children with selective eating habits. Brain and Development, 42(2), 121–128. https://doi.org/10.1016/j.braindev.2019.09.005sis, 35(3), 259-270. https://doi.org/10.1901/jaba.2002.35-259
